# Oxygen Nonstoichiometry, Electrical Conductivity, Chemical Expansion and Electrode Properties of Perovskite-Type SrFe_0.9_V_0.1_O_3−δ_

**DOI:** 10.3390/ma18030493

**Published:** 2025-01-22

**Authors:** Aleksei I. Ivanov, Sergey S. Nikitin, Mariya S. Dyakina, Ekaterina V. Tsipis, Mikhail V. Patrakeev, Dmitrii A. Agarkov, Irina I. Zverkova, Andrey O. Zhigachev, Victor V. Kedrov, Vladislav V. Kharton

**Affiliations:** 1 Osipyan Institute of Solid State Physics RAS, Chernogolovka 142432, Russia; nikitin@issp.ac.ru (S.S.N.); dyakina@issp.ac.ru (M.S.D.); patrakeev@issp.ac.ru (M.V.P.); agarkov@issp.ac.ru (D.A.A.); zverkova@issp.ac.ru (I.I.Z.); zhigachev@issp.ac.ru (A.O.Z.); kedr@issp.ac.ru (V.V.K.); kharton@issp.ac.ru (V.V.K.); 2 Moscow Institute of Physics and Technology, Dolgoprudny 141701, Russia

**Keywords:** vanadium-doped strontium ferrite, defect structure, electrical conductivity, thermal expansion, chemical expansion, SOFC/SOEC electrodes

## Abstract

X-ray diffraction analysis of the pseudo-binary SrFe_1−*x*_V*_x_*O_3−δ_ system showed that the solid solution formation limit at atmospheric oxygen pressure corresponds to *x* ≈ 0.1. SrFe_0.9_V_0.1_O_3−δ_ has a cubic perovskite-type structure with the *Pm*$\bar{3}$*m* space group. The oxygen nonstoichiometry variations in SrFe_0.9_V_0.1_O_3−δ_, measured by coulometric titration in the oxygen partial pressure range of 10^−21^ to 0.5 atm at 1023–1223 K, can be adequately described using an ideal solution approximation with V^5+^ as the main oxidation state of vanadium cations. This approach was additionally validated by statistical thermodynamic modeling. The incorporation of vanadium decreases both oxygen deficiency and the average iron oxidation state with respect to undoped SrFeO_3−δ_. As a result, the electrical conductivity, thermal expansion and chemical expansivity associated with the oxygen vacancy formation all become lower compared to strontium ferrite. At 923 K, the conductivity of SrFe_0.9_V_0.1_O_3−δ_ is 14% lower than that of SrFeO_3−δ_ but 21% higher compared to SrFe_0.9_Ta_0.1_O_3−δ_. The area-specific polarization resistance of the porous SrFe_0.9_V_0.1_O_3−δ_ electrode deposited onto 10 mol.% scandia- and 1 mol.% yttria-co-stabilized zirconia solid electrolyte with a protective Ce_0.9_Gd_0.1_O_2−δ_ interlayer, was 0.34 Ohm×cm^2^ under open-circuit conditions at 1173 K in air.

## 1. Introduction

In recent years, structural features and transport properties of perovskite-type strontium ferrite (SrFeO_3−δ_) have attracted significant attention due to its wide range of oxygen deficiency (2.5 ≤ (3 − δ) ≤ 3) and high mixed ionic–electronic conductivity [1,2,3,4,5,6,7]. However, the practical applications of SrFeO_3−δ_ in high- and intermediate-temperature electrochemical devices are limited by several factors. First, an increase in oxygen nonstoichiometry under reducing conditions leads to phase transition into orthorhombic brownmillerite, which is accompanied by a substantial decrease in conductivity and large volume changes [1,3,4]. Second, the oxygen release from an SrFeO_3−δ_ crystal lattice on heating results in a drastic increase in the apparent average thermal expansion coefficient (TEC) due to the so-called chemical contribution [2,3,7,8]. Third, at atmospheric oxygen pressure, the cubic structure of SrFeO_3−δ_ perovskite only exists at elevated temperatures; its tetragonal polymorph is formed on cooling down to room temperature, again with volume changes [2,3,8]. Notice that the cubic vacancy-disordered structure of SrFeO_3−δ_ with the space group (SG) *Pm*$\bar{3}$*m* provides the highest level of mixed conductivity due to the isotropic transport of electronic and ionic charge carriers [4,8].

Another material to attract attention is perovskite-type strontium vanadate (SrVO_3−δ_). This phase exhibits moderate apparent TEC and high electronic conductivity under reducing conditions, namely ~10^3^ S/cm at the oxygen partial pressure (*p*(O_2_)) of 10^−20^ atm [9,10,11,12]. SrVO_3−δ_ and its La-substituted analogues are widely considered as anode materials for solid oxide fuel cells (SOFCs) using both hydrogen and cheaper hydrocarbon-based fuels; in particular, those containing H_2_S and other harmful impurities [13,14]. The main disadvantage of SrVO_3−δ_ relates to phase instability on redox cycling [10,12,15]. When SrVO_3−δ_ is annealed in air, phase decomposition leads to a decrease in electrical conductivity by approximately six orders of magnitude and to significant volume expansion [10,12,15]. Moreover, this decomposition may be irreversible [10].

Taking into account the advantages of SrFeO_3_- and SrVO_3_-based materials, the present work was centered on studies of the pseudo-binary SrFe_1−*x*_V*_x_*O_3−δ_ system. Particular attention was paid to the functional properties important for the electrodes of SOFCs and solid oxide electrolysis cells (SOECs). Doping of SrFeO_3−δ_ with higher-valency cations (e.g., Nb, Ta, Mo or W) having predominant octahedral coordination with oxygen is well known to stabilize the cubic perovskite structure in a wide temperature range, to suppress the “perovskite → brownmillerite” transition under reducing conditions, and to decrease apparent TECs [5,6,7,16,17,18,19,20]. High-resolution electron microscopic analysis of SrFe_1−*x*_V*_x_*O_3−δ_, synthesized in an evacuated quartz tube with quenching from 1473 K, showed that vanadium doping does indeed have a noticeable effect on the structure, even at low dopant concentrations [21]. Namely, although the formation of nanodomains (≤20 nm) with a brownmillerite structure was observed in the compositional range of 0.05 ≤ *x* ≤ 0.10, the presence of vanadium and excess oxygen blocked nanodomain growth [21]. Similar conclusions have been drawn for SrFe_1−*x*_V*_x_*O_3−δ_ (0 ≤ *x* ≤ 0.08) synthesized in a vacuum followed by quenching from 1173–1223 K [22,23]. The formation of ALaFeVO_6_ (A = Ca, Sr) double perovskites was achieved by annealing in evacuated and sealed silica tubes at 1273 K for 8 days [24]. Such synthetic approaches cannot, however, be effective for SOFC/SOEC technologies, as each porous electrode in these devices is unavoidably equilibrated with the relevant atmosphere at elevated temperatures. In the present work, synthesis of SrFe_1−*x*_V*_x_*O_3−δ_ was performed in atmospheric air.

## 2. Materials and Methods

The synthesis of SrFe_1−*x*_V*_x_*O_3−δ_ (*x* = 0.10, 0.15 and 0.20) powders was carried out by the citrate–nitrate method using Sr(NO_3_)_2_, FeC_2_O_4_·2H_2_O, NH_4_VO_3_ and C_6_H_8_O_7_·H_2_O (≥99% purity) as starting reagents. Notice that combustion synthesis routes are often used for commercial-scale applications. At the initial stage of the synthesis, Sr(NO_3_)_2_, NH_4_VO_3_ and C_6_H_8_O_7_·H_2_O were dissolved in distilled water with vigorous stirring; citric acid was taken in threefold excess with respect to the metal cations introduced in the stoichiometric ratio. The stoichiometric amount of iron oxalate was separately dissolved in dilute nitric acid. Then, the solutions were mixed and evaporated. Further smoldering of the mixtures resulted in fine powders, which were calcined in air at 1173 K for 2 h to remove the organic residues. Following careful grinding, the final annealing step was carried out in air during 10 h at 1373–1473 K with a heating/cooling rate of 3 K/min. The synthesized powders were ball-milled in ethanol media at 400 rpm for 2 h using a Fritsch planetary ball mill with containers and balls made of partially stabilized zirconia. Ceramic samples were obtained by uniaxial pressing in the form of disks (diameter of 20 mm and thickness of 2–3 mm) at ~200 MPa and sintered in air at 1553 K for 2 h. For the studies of transport and thermomechanical properties, rectangular bars (2 × 2 × 10 mm^3^) were cut from the sintered disks. The microstructure of the powders, ceramics and model electrochemical cells was analyzed using scanning electron microscopy (SEM, LEO SUPRA 50VP, Carl Zeiss, Oberkochen, Germany) combined with energy-dispersive X-ray spectroscopy (EDX) analyses.

X-ray diffraction (XRD) analysis was performed at room temperature on a Rigaku SmartLab SE diffractometer (CuK_α_ radiation, 20° ≤ 2θ ≤ 80°). Phase analysis of the samples and calculation of lattice parameters were performed using Match and PowderCell (version 2.4) software. The particle size distributions of the powders were assessed by static laser light scattering (Analysette 22 Next Nano, Fritsch, Luxemburg, Luxemburg). Prior to the analysis, the powders were dispersed in bi-distilled water employing ultrasonic radiation. In order to evaluate stability under reducing conditions, powdered SrFe_1−*x*_V*_x_*O_3−δ_ samples were annealed in a 4% H_2_-Ar-H_2_O gas mixture where the oxygen partial pressure was controlled using a zirconia electrochemical sensor. Thermogravimetric analysis (TGA) was carried out in flowing atmospheric air using a Setaram Setsys EVO 16 instrument (Setaram, Caluire, France); the measurements were performed in a cooling mode (1 K/min) after equilibration at 1223 K for 5 h. The electrical conductivity was measured by the 4-probe DC technique at 473–1223 K in air and Ar flows.

The variations of oxygen nonstoichiometry were studied by coulometric titration in a *p*(O_2_) range of 10^−21^–0.5 atm at 1023–1223 K with a step of 50 K in the CO_2_-CO-O_2_ gas system. The measurements were performed using a double electrochemical cell, equipped with an oxygen pump and a sensor, made of stabilized zirconia with porous Pt electrodes. The experimental data points were recorded after equilibration of the sample with the gas phase. The equilibrium criteria included the rate of changes in the logarithm of *p*(O_2_) less than 10^−4^ per minute and the standard deviation from a mean value lower than 0.03% at fixed temperature. The reliability of the collected data was evaluated using reproducibility analysis in the course of thermo or redox cycling. The measurement procedures and equipment were detailed elsewhere [5].

Thermal expansion of the ceramic materials in air and argon was tested using a Linseis L75VS1400 vertical alumina dilatometer (Linseis, Selb, Germany). The measurements were conducted in dynamic and static regimes. In dynamic mode, the sample was continuously heated at a rate of 3 K/min up to 1273 K, equilibrated for 2 h at this temperature and then cooled down to 373 K at the same rate. In static mode, the sample was heated up to 1273 K, followed by stepwise cooling down to 873 K (step 50 K) with isothermal equilibration dwells at each temperature. Examples illustrating this measuring mode for SrFe_0.9_V_0.1_O_3−δ_ are presented in Electronic Supplementary Materials (Figure S1). The isothermal chemical expansion (ε_chem_) on “air → argon” transition at temperature *T* was calculated by the equation

(1) $$\varepsilon_{\mathrm{chem}}=\frac{L_{\mathrm{Ar},T}-L_{\mathrm{air},T}}{L_{\mathrm{air},T}}$$

where *L*_Ar,*T*_ and *L*_air,*T*_ are the sample length values in argon and air, respectively.

The electrochemical tests of porous electrode layers were carried out by the 3-electrode technique, as reported elsewhere [25,26]. The model half-cells comprised one gas-tight disk (1 mm thick) of 10 mol.% scandia- and 1 mol.% yttria-co-stabilized zirconia solid electrolyte (10Sc1YSZ, DKKK, Osaka, Japan), a working electrode (WE) of porous SrFe_1−*x*_V*_x_*O_3−δ_ deposited onto a protective Ce_0.9_Gd_0.1_O_2−δ_ (GDC10, Kceracell, Geumsan-gun, Republic of Korea) interlayer, and porous Pt counter and reference electrodes (CE and RE, respectively). The oxide layers were made of inks containing ball-milled powders, V-006A binder (Heraeus GmbH, Hanau, Germany) and terpineol. The protective GDC10 interlayer (semicircular with a geometric area of 1.2 cm^2^) was first screen-printed onto one side of the electrolyte membrane, followed by sintering at 1473 K for 2 h. Then GDC10 was screen-printed again and, after drying at 373 K, the WE layer was deposited. The protective interlayer and WE were sintered in air at 1373 K for 2 h with a heating/cooling rate of 4 K/min. The CE, symmetrically with respect to the WE, was applied onto the opposite side of the solid electrolyte. The RE (~1 mm in diameter) was placed at a distance of at least 6 mm from the WE. The platinum CE and RE were annealed in air at 1223 K for 30 min. A Pt mesh and Pt wire were used as current collectors for the WE and CE, respectively. Neither additional layers nor other noble metals were applied on the WE surface in order to avoid possible contributions to its electrochemical performance. The measurements were performed using an AutoLab PGSTAT302N potentiostat/galvanostat (Metrohm, Utrecht, The Netherlands) at 1023–1173 K in static air. The relaxation time after a change in the WE potential was 60 min. Ohmic and polarization resistance values were extracted from impedance spectra collected in a frequency range from 1 MHz down to 0.1 Hz.

## 3. Results and Discussion

### 3.1. Phase Relationships and Crystal Structure

**Figure 1:**
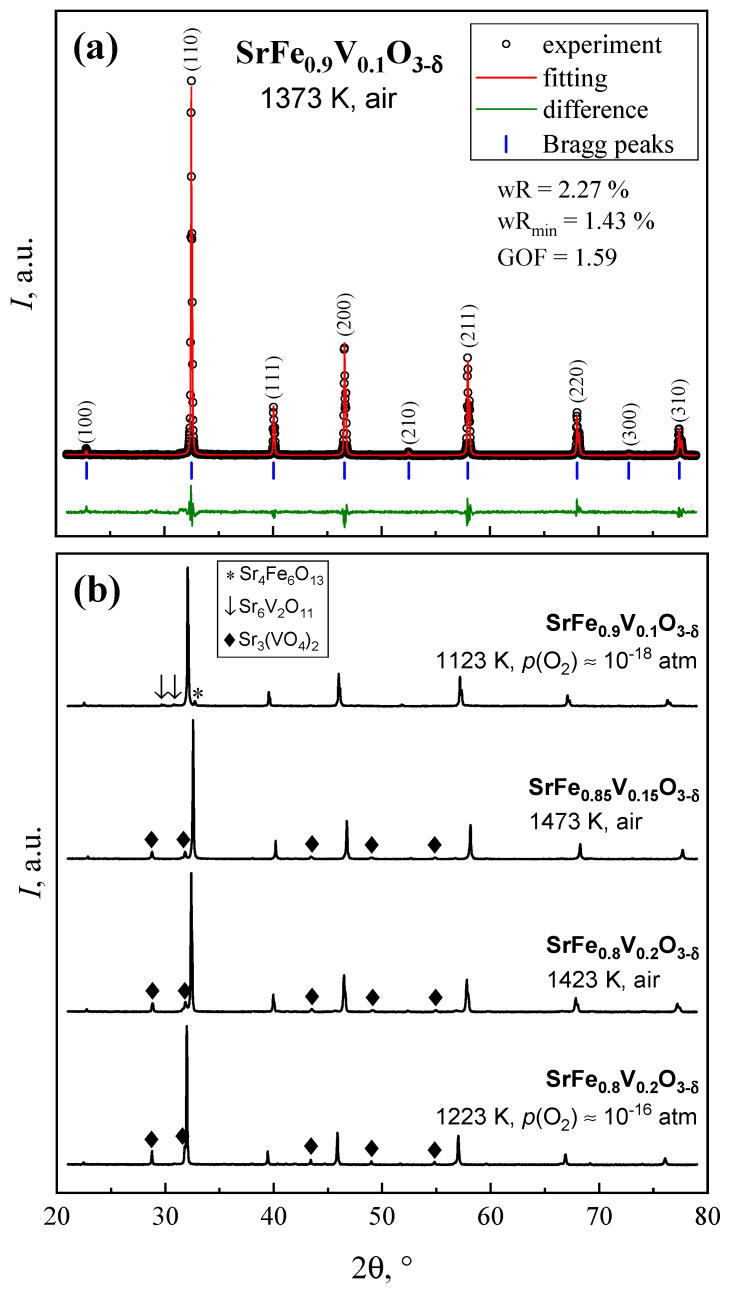
Room temperature XRD pattern of SrFe_0.9_V_0.1_O_3−δ_ powder after synthesis in air (**a**) and of SrFe_1−*x*_V*_x_*O_3−δ_ powders after synthesis in air and annealing in reducing conditions (**b**).

XRD patterns of SrFe_1−*x*_V*_x_*O_3−δ_ (*x* = 0.10–0.20) synthesized in air are presented in Figure 1. The composition with *x* = 0.1 is single phase and has a cubic perovskite-type structure (SG *Pm*$\bar{3}$*m*) and a unit cell parameter *a* = 3.8947 ± 0.0003 Å. Note that, for undoped SrFeO_3−δ_ prepared in the same conditions [5], a tetragonal polymorph with SG *I*4/*mmm* is formed. Reduction of SrFe_0.9_V_0.1_O_3−δ_ in a flow of 4% H_2_-Ar-H_2_O gas mixture at 1123 K and *p*(O_2_) ≈ 10^−18^ atm results in the segregation of Sr_4_Fe_6_O_13_ and Sr_6_V_2_O_11_ impurities. This behavior indicates that, although the incorporation of 10% vanadium in the iron sublattice of strontium ferrite stabilizes the cubic perovskite phase down to room temperature in air, the thermodynamic stability of SrFe_0.9_V_0.1_O_3−δ_ under reducing conditions is insufficient for use as an SOFC anode or SOEC cathode material, even in mild reducing atmospheres. At the same time, the latter composition is close to the solid solution formation limit in an SrFe_1−*x*_V*_x_*O_3−δ_ system. In the case of *x* = 0.15–0.20, the peaks of Sr_3_(VO_4_)_2_ impurity are visible in the XRD patterns, either after synthesis in air or after reduction at 1223 K and *p*(O_2_) ≈ 10^−16^ atm. Consequently, SrFe_0.85_V_0.15_O_3−δ_ and SrFe_0.8_V_0.2_O_3−δ_ were excluded from further studies.

### 3.2. Microstructure

**Figure 2:**
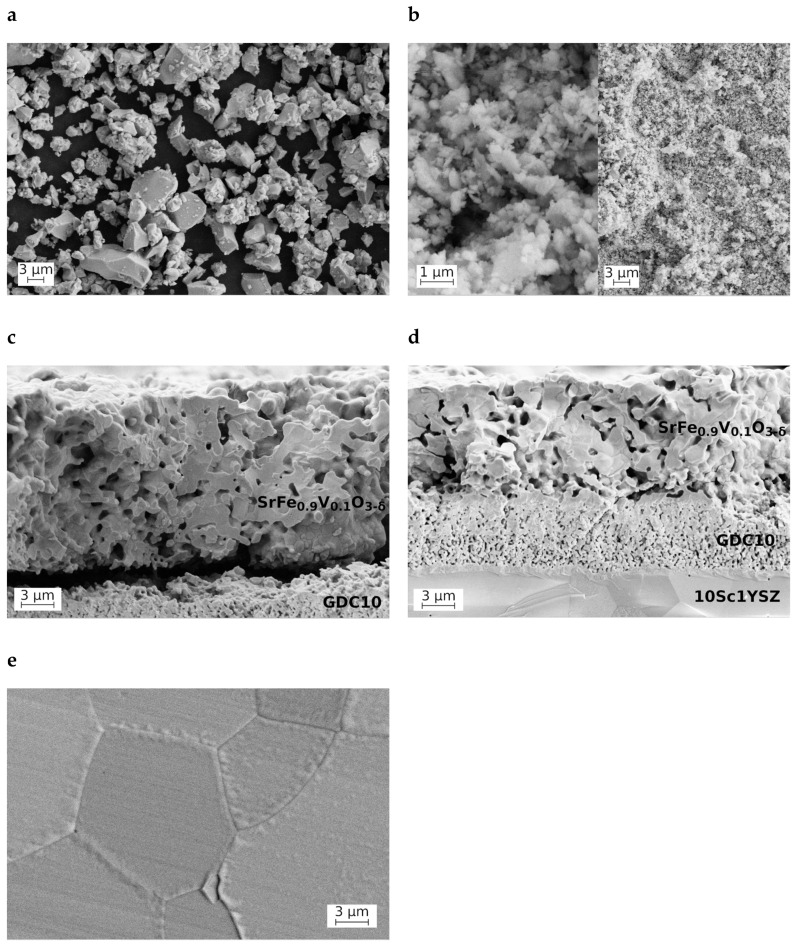
SEM micrographs of SrFe_0.9_V_0.1_O_3−δ_ powder as-synthesized (**a**) and after ball-milling (**b**), and porous electrode layers (cross-sections of the electrochemical cells) made of as-synthesized (**c**) and milled (**d**) powders sintered in air at 1473 K, and dense ceramics sintered at 1553 K (**e**).

**Figure 3:**
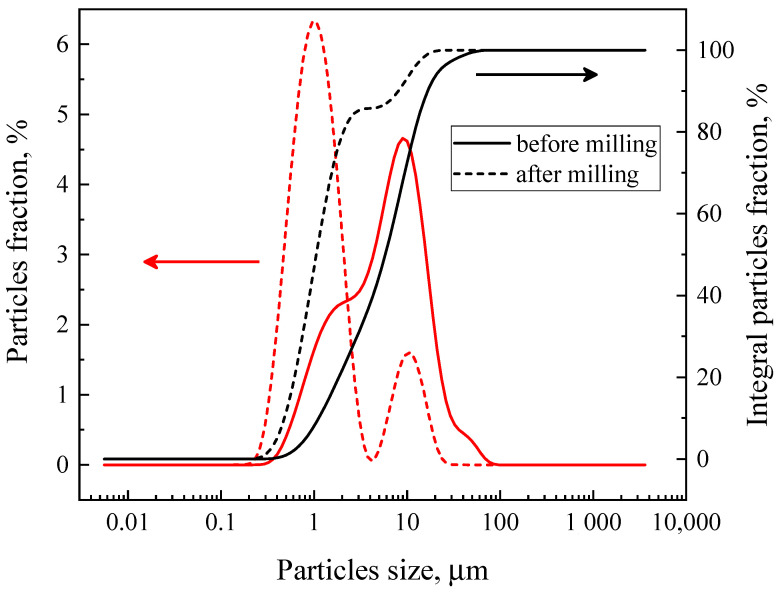
Particle size distributions in the initial and ground powders of SrFe_0.9_V_0.1_O_3−δ_, determined by the static laser radiation scattering method.

A typical SEM micrograph illustrating the powder morphology after final annealing in air at 1473 K is shown in Figure 2a. The powder consists of submicron and micron-size particles and is highly agglomerated. Static laser scattering (Figure 3) shows that the majority of the agglomerates are fairly large, with an average of 9 μm. There are also significant amounts of small agglomerates or individual particles of approximately 2 μm in size and a small number of large 40 μm agglomerates. The electrode layers made of this powder possess a low porosity and poor adhesion to the protective GDC10 interlayer (Figure 2c). Therefore, in order to prepare inks for screen printing the electrode layers used for the electrochemical measurements, the powders were milled. The resultant powder consists of fine submicron particles (Figure 2b) forming small agglomerates with average sizes of about 1 μm and 10 μm with an approximate ratio of 4:1 (Figure 3).

Figure 2d displays cross-sections of the electrochemical cell comprising 10Sc1YSZ solid electrolyte, protective GDC10 interlayer, and SrFe_0.9_V_0.1_O_3−δ_ working electrode. The ceria-based interlayer with a thickness of ~6 μm shows good adhesion to the dense electrolyte membrane and to the WE. The porous SrFe_0.9_V_0.1_O_3−δ_ electrode is built of micron-scale particles (0.5–2 μm in size) and has a thickness of ~11 μm. The cell with this microstructure was selected for further studies.

Figure 2e illustrates the microstructure of SrFe_0.9_V_0.1_O_3−δ_ ceramics used for thermomechanical and electrical measurements. The grain size varied in the range of 4–10 μm. No visible inhomogeneities were detected by SEM/EDX analyses.

### 3.3. Oxygen Nonstoichiometry and Phase Stability Limit

**Figure 4:**
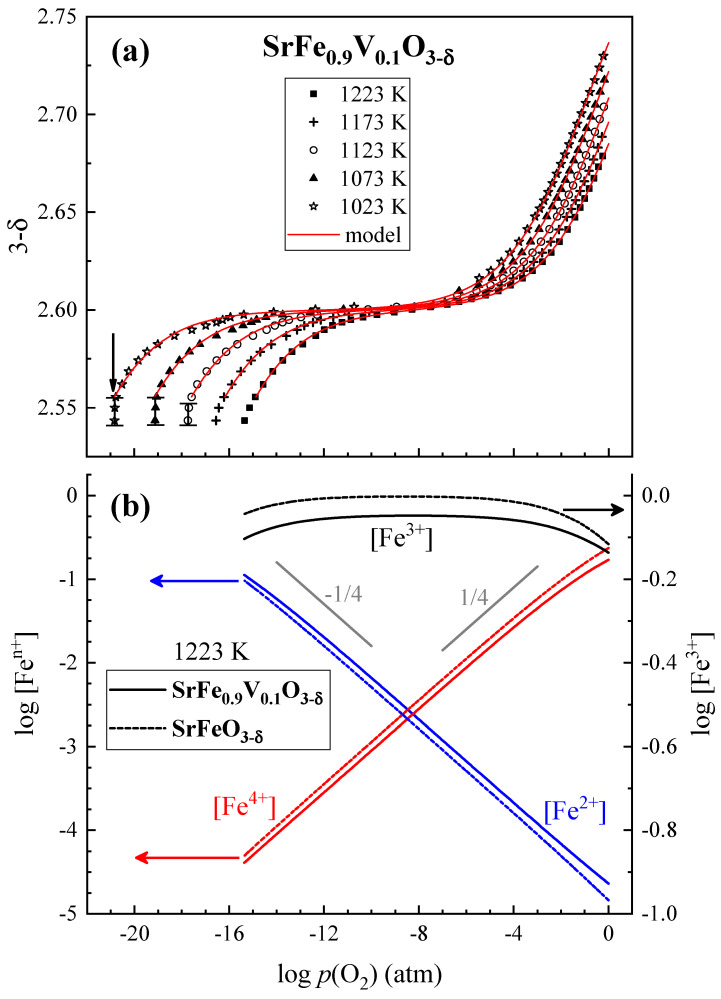
Oxygen partial pressure dependencies of the oxygen content (**a**) and the calculated concentrations of iron cations in different oxidation states (**b**). For (**a**), red solid lines correspond to the fitting results and vertical line segments indicate phase decomposition. The data on SrFeO_3−δ_ [5] are shown for comparison.

The *p*(O_2_)-*T*-(3-δ) diagram of SrFe_0.9_V_0.1_O_3−δ_ (Figure 4a) is typical for most perovskite-type solid solutions based on strontium ferrite [5,26]. In oxidizing atmospheres, the oxygen content decreases with decreasing *p*(O_2_) as Fe^4+^ states are progressively reduced to Fe^3+^. Then, in the intermediate *p*(O_2_) range, the isotherms exhibit inflection points. At lower *p*(O_2_), the oxygen deficiency continues to increase on reduction, which is associated with the appearance of Fe^2+^ and, possibly, V^4+^ states. The *p*(O_2_)-*T*-(3-δ) diagram also displays clear indications of perovskite phase decomposition under reducing conditions at 1023–1123 K. The decomposition is reflected by *p*(O_2_)-independent variations in the oxygen content, marked by the vertical line segments in Figure 4a. The corresponding oxygen partial pressures can be considered to be the perovskite phase stability limits.

**Figure 5:**
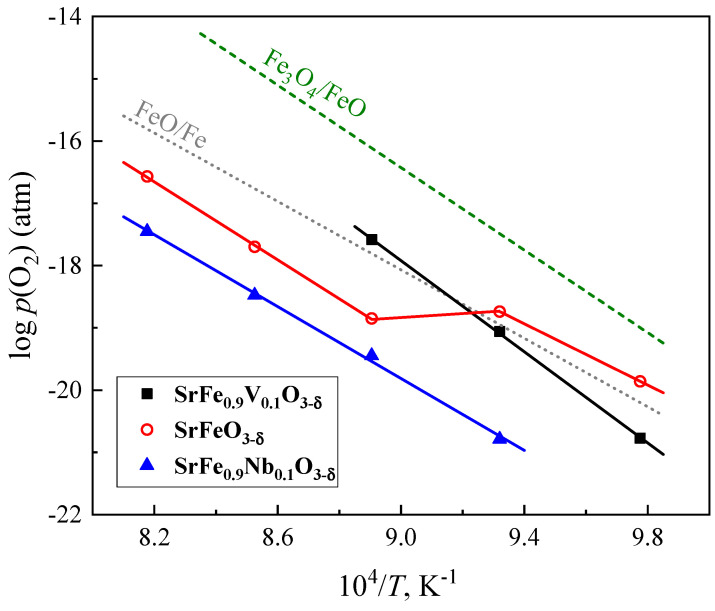
Stability limit of SrFe_0.9_V_0.1_O_3−δ_ perovskite under reducing conditions. The data on SrFeO_3−δ_ [4], SrFe_0.9_Nb_0.1_O_3−δ_ [17], and Fe_3_O_4_/FeO and FeO/Fe phase boundaries [27] are shown for comparison.

Figure 5 compares the stability boundary of SrFe_0.9_V_0.1_O_3−δ_ with data from the literature on SrFeO_3−δ_ [4], SrFe_0.9_Nb_0.1_O_3−δ_ [17] and binary iron oxides [27]. The thermodynamic stability of perovskite-type SrFe_0.9_V_0.1_O_3−δ_ is moderately higher than that of undoped strontium ferrite at temperatures below 1073 K when the latter possesses a brownmillerite structure. At higher temperatures, when a brownmillerite polymorph transforms into cubic SrFeO_3−δ_ perovskite, the stability of SrFe_0.9_V_0.1_O_3−δ_ with respect to reductive decomposition is lower. The stability of SrFe_0.9_V_0.1_O_3−δ_ is also worse than that of the Nb-containing analogue (Figure 5). As mentioned above, the title composition may hardly be used for SOFC anodes and SOEC cathodes. At the same time, its stability under reducing conditions may be improved by the partial substitution of Sr for rare-earth cations.

### 3.4. Point Defect Formation Model

Within the perovskite phase existence domain, the *p*(O_2_)-*T*-(3-δ) diagram of SrFe_0.9_V_0.1_O_3−δ_ can be described by a point defect model similar to those used for other SrFeO_3_ and La_1−x_Sr_x_FeO_3−δ_-based solid solutions [5,26,28,29,30]. This model assumes dominant roles for three major defect reactions, namely, iron oxidation (Equation (2)), charge disproportionation of iron cations (Equation (3)), and electron exchange between iron and vanadium ions (Equation (4)):

(2) $$\frac{1}{2}O_{2}+V_{O}+2{\mathrm{Fe}}^{3+}⇄O^{2-}+2{\mathrm{Fe}}^{4+},\;K_{\mathrm{ox}}=\frac{\left[O^{2-}\right]{\left[{\mathrm{Fe}}^{4+}\right]}^{2}}{p{\left(O_{2}\right)}^{0.5}\left[V_{O}\right]{\left[{\mathrm{Fe}}^{3+}\right]}^{2}};$$

(3) $$2{\mathrm{Fe}}^{3+}⇄{\mathrm{Fe}}^{4+}+{\mathrm{Fe}}^{2+},\;K_{d}=\frac{\left[{\mathrm{Fe}}^{4+}\right]\left[{\mathrm{Fe}}^{2+}\right]}{{\left[{\mathrm{Fe}}^{3+}\right]}^{2}};$$

(4) $${\mathrm{Fe}}^{3+}+V^{4+}⇄{\mathrm{Fe}}^{2+}+V^{5+},\;K_{e}=\frac{\left[{\mathrm{Fe}}^{2+}\right]\left[V^{5+}\right]}{\left[{\mathrm{Fe}}^{3+}\right]\left[V^{4+}\right]},$$

where *K*_ox_, *K*_d_ and *K*_e_ are the corresponding equilibrium constants. This model is based on the ideal solution approximation when the standard thermodynamic functions of the reactions (2)–(4) are independent of defect concentrations, leading to the following temperature dependence of all equilibrium constants:

(5) $$K_{i}=\exp\left(-\frac{\Delta G_{i}^{0}}{RT}\right)=\exp\left(-\frac{\Delta H_{i}^{0}}{RT}+\frac{\Delta S_{i}^{0}}{R}\right),$$

where *R* is the gas constant, whereas $\Delta G_{i}^{0}$, $\Delta H_{i}^{0}$, $\Delta S_{i}^{0}$ are the standard free Gibbs energy, enthalpy and entropy for the defect formation reactions, respectively. The site conservation conditions were formulated as

(6) $$\{\begin{array}{l} \left[{\mathrm{Fe}}^{2+}\right]+\left[{\mathrm{Fe}}^{3+}\right]+\left[{\mathrm{Fe}}^{4+}\right]=0.9\\ \left[V^{4+}\right]+\left[V^{5+}\right]=0.1 \end{array},$$

(7) $$\{\begin{array}{l} \left[O^{2-}\right]=3-\delta -\Delta \delta_{\mathrm{ref}}\\ \left[V_{O}\right]=\delta -w-\Delta \delta_{\mathrm{ref}} \end{array}.$$

Equation (7) contains two empirical parameters, Δδ_ref_ and *w*. The former, reflecting experimental errors, is introduced to correct experimental data on the oxygen content. The *w* parameter corresponds to the number of anion sites unavailable for oxygen ions for various physical reasons, such as phenomena related to local ordering and grain boundaries, per formula unit. Finally, the electroneutrality condition can be written as follows:

(8) $$2\left[{\mathrm{Sr}}^{2+}\right]+2\left[{\mathrm{Fe}}^{2+}\right]+3\left[{\mathrm{Fe}}^{3+}\right]+4\left[{\mathrm{Fe}}^{4+}\right]+4\left[V^{4+}\right]+5\left[V^{5+}\right]=2\left[O^{2-}\right].$$

Solving of Equations (2)–(8) makes it possible to formulate the following final model:

(9) $$5.2-0.9\frac{K_{d}-K_{\mathrm{ox}}T_{s}}{K_{d}+K_{\mathrm{ox}}T_{s}+\sqrt{K_{\mathrm{ox}}T_{s}}}-0.1\frac{K_{d}}{K_{d}+K_{e}\sqrt{K_{\mathrm{ox}}T_{s}}}=2\left(3-\delta +\Delta \delta_{\mathrm{ref}}\right),$$

where $T_{s}=\frac{\delta -w-{\Delta \delta }_{\mathrm{ref}}}{3-\delta +{\Delta \delta }_{\mathrm{ref}}}\cdot \sqrt{p(O_{2})}$. The Levenberg–Marquardt algorithm of the LMFIT library [31] was used for fitting Equation (9) to the experimental data.

Preliminary calculations demonstrated that the thermodynamic parameters of the electron exchange reaction between iron and vanadium ($\Delta H_{e}^{0}$ and $\Delta S_{e}^{0}$) have high rates of error, whilst their variations have no effect on the fitting results. Therefore, the model was simplified by excluding Equation (4). In other words, the presence of V^4+^ was neglected in Equations (6) and (8); V^5+^ concentration was assumed to be constant and equal to 0.1.

The fitting results using this model (red solid lines in Figure 4a) well describe the experimental data. Table 1 compares the thermodynamic parameters calculated for SrFe_0.9_V_0.1_O_3−δ_ and those for similar materials reported elsewhere. Within the uncertainty limits, thermodynamic functions of the oxidation and iron disproportionation reactions are similar for all presented compositions, except for undoped SrFeO_3−δ_.

### 3.5. Defect Concentrations and Oxygen Thermodynamic Functions

The obtained thermodynamic parameters were used to calculate concentrations of iron states as a function of *p*(O_2_). As an example, Figure 4b compares the variations for SrFe_0.9_V_0.1_O_3−δ_ and SrFeO_3−δ_ at 1223 K. Under moderately oxidizing and reducing conditions, the concentrations of Fe^2+^ and Fe^4+^ are proportional to *p*(O_2_)^±1/4^. At high oxygen pressures, when the average oxidation state of iron cations is far from 3+, a deviation from the power law is observed. The calculations showed that the introduction of vanadium into the iron sublattice of SrFeO_3−δ_ increases Fe^2+^ content and decreases Fe^4+^ concentration with respect to undoped ferrite.

The experimental *p*(O_2_)-*T*-(3-δ) diagram was then used to calculate the increments of oxygen chemical potential (∆μ_O_):

(10) $$\Delta \mu_{O}=\frac{1}{2}RT\ln p\left(O_{2}\right).$$

As the dependence of ∆μ_O_ on temperature at fixed (3 − δ) values is linear, the partial molar enthalpy (∆*h*_O_) and entropy (∆*s*_O_) of oxygen incorporation in the lattice can be extracted:

(11) $$\Delta \mu_{O}=\Delta h_{O}-T\Delta s_{O}.$$

**Figure 6:**
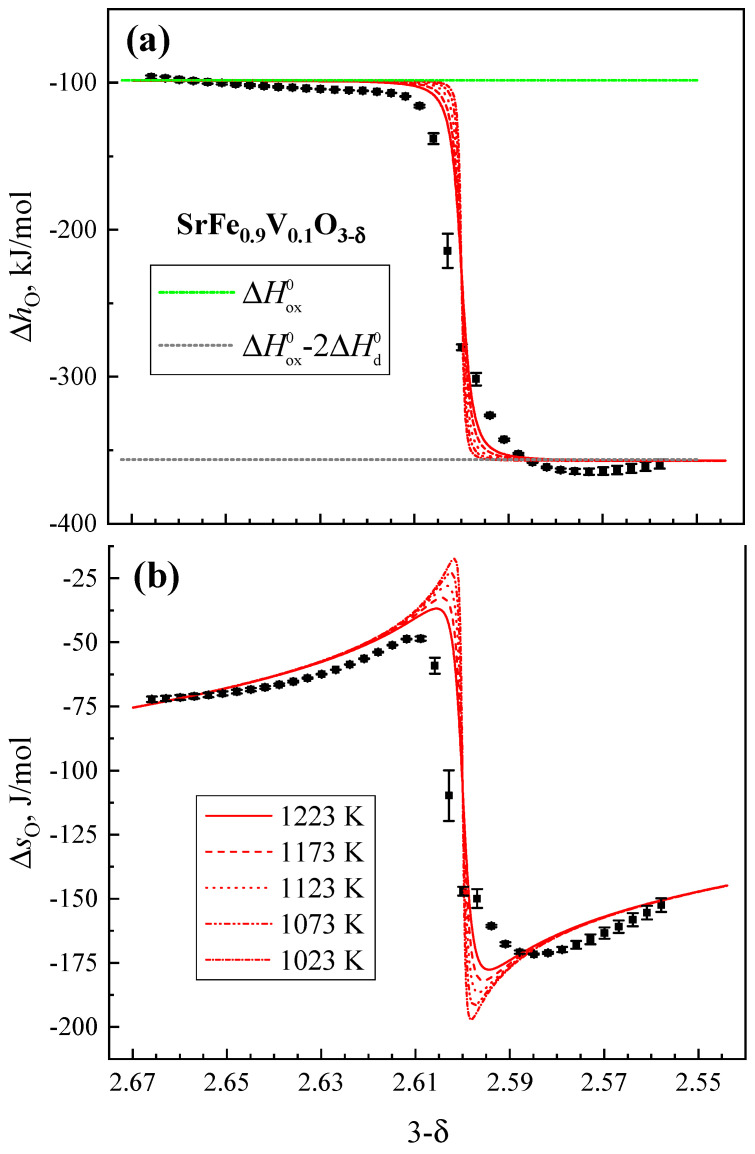
Partial molar enthalpy (**a**) and entropy (**b**) of oxygen as functions of the oxygen content in SrFe_0.9_V_0.1_O_3−δ_. Dots present the results obtained using the Gibbs–Helmholtz relationship. Red lines show the results of statistical thermodynamic modeling.

The calculated values of the partial molar thermodynamic functions of oxygen are shown by dots in Figure 6a,b). Their dependencies on the oxygen vacancy concentration are non-monotonic and have breakpoints at (3 − δ) ≈ 2.6 associated with changing dominant mechanisms of the defect formation, as discussed below. The partial molar enthalpy is almost constant before and after its breakpoint (Figure 6a), thus confirming the applicability of the ideal solution approximation. It should be noted, however, that this approximation may not always be applicable in complex oxide systems, especially in systems involving multiple cations and substantial variations in oxygen nonstoichiometry. For instance, a deviation from the ideal solution behavior in undoped SrFeO_3−δ_ is observed near δ = 0.5, reflected by a decrease in the partial molar entropy of oxygen due to the vacancy ordering [32].

### 3.6. Statistical Thermodynamic Modeling

In order to additionally verify the adequacy of the calculated thermodynamic parameters listed in Table 1, these were used for statistical thermodynamic modeling of the partial molar enthalpy and entropy. The modeling results were then compared to the quantities directly extracted from the experimental *p*(O_2_)-*T*-(3-δ) diagram.

Using the ideal solution approximation and assuming statistical distribution of all defect species in the corresponding sublattices, the total Gibbs energy for SrFe_0.9_V_0.1_O_3−δ_ is determined as [30]

(12) $$G=G^{0}+\left[{\mathrm{Sr}}^{2+}\right]\mu_{{\mathrm{Sr}}^{2+}}^{0}+\left[{\mathrm{Fe}}^{2+}\right]\mu_{{\mathrm{Fe}}^{2+}}^{0}+\left[{\mathrm{Fe}}^{3+}\right]\mu_{{\mathrm{Fe}}^{3+}}^{0}+\left[{\mathrm{Fe}}^{4+}\right]\mu_{{\mathrm{Fe}}^{4+}}^{0}+\left[V^{5+}\right]\mu_{V^{5+}}^{0}+\left(3-\delta \right)\mu_{O^{2-}}^{0}+{\delta \mu }_{V_{O}}^{0}-T\cdot S_{\mathrm{conf}},$$

where $G^{0}$ is the standard Gibbs energy and $S_{\mathrm{conf}}$ is the total configurational entropy. The latter quantity in the Stirling approximation is defined by the formula [33]

(13) $$S_{\mathrm{conf}}=-R\cdot \sum_{i}x_{i}\ln\left(x_{i}\right),$$

where $x_{i}$ is the concentration of *i*-th component (Sr^2+^, Fe^2+^, Fe^3+^, Fe^4+^, V^5+^, O^2−^, V_O_). The chemical potential of oxygen can be determined as

(14) $$\mu_{O}=\frac{\partial G}{\partial \left[O^{2-}\right]}=\frac{\partial G}{\partial \left(3-\delta \right)}=-\frac{\partial G}{\partial \delta }=-\frac{\partial \left[{\mathrm{Fe}}^{2+}\right]}{\partial \delta }\cdot \mu_{{\mathrm{Fe}}^{2+}}^{0}-\frac{\partial \left[{\mathrm{Fe}}^{3+}\right]}{\partial \delta }\cdot \mu_{{\mathrm{Fe}}^{3+}}^{0}-\frac{\partial \left[{\mathrm{Fe}}^{4+}\right]}{\partial \delta }\cdot \mu_{{\mathrm{Fe}}^{4+}}^{0}+\mu_{O^{2-}}^{0}-\mu_{V_{O}}^{0}-T\cdot s_{O\;\mathrm{conf}},$$

(15) $$s_{O\;\mathrm{conf}}=-\frac{\partial S_{\mathrm{conf}}}{\partial \delta }.$$

Using Equations (2)–(8) and (13)–(15), one can obtain

(16) $${\Delta \mu }_{O}=\left(\Delta G_{\mathrm{ox}}^{0}-2\cdot \Delta G_{d}^{0}\right)-\frac{\partial \left[{\mathrm{Fe}}^{4+}\right]}{\partial \delta }\cdot \Delta G_{d}^{0}-T\cdot s_{O\;\mathrm{conf}},$$

(17) $$\Delta h_{O}=\left(\Delta H_{\mathrm{ox}}^{0}-2\cdot \Delta H_{d}^{0}\right)-\frac{\partial \left[{\mathrm{Fe}}^{4+}\right]}{\partial \delta }\cdot \Delta H_{d}^{0},$$

(18) $$\Delta s_{O}=\left(\Delta S_{\mathrm{ox}}^{0}-2\cdot \Delta S_{d}^{0}\right)-\frac{\partial \left[{\mathrm{Fe}}^{4+}\right]}{\partial \delta }\cdot \Delta S_{d}^{0}+s_{O\;\mathrm{conf}},$$

(19) $$s_{O\;\mathrm{conf}}=R\left(\frac{\partial \left[{\mathrm{Fe}}^{4+}\right]}{\partial \delta }\cdot \ln\left(K_{d}\right)+\ln\left(\frac{{K_{d}}^{2}}{K_{\mathrm{ox}}\cdot \sqrt{p_{O_{2}}}}\right)\right).$$

For the concentrations of Fe^2+^, Fe^3+^, Fe^4+^, oxygen ions and vacancies

(20) $$\left[{\mathrm{Fe}}^{2+}\right]=\frac{0.9\cdot K_{d}}{K_{\mathrm{ox}}T_{s}+\sqrt{K_{\mathrm{ox}}T_{s}}+K_{d}},$$

(21) $$\left[{\mathrm{Fe}}^{3+}\right]=\frac{0.9\cdot \sqrt{K_{\mathrm{ox}}T_{s}}}{K_{\mathrm{ox}}T_{s}+\sqrt{K_{\mathrm{ox}}T_{s}}+K_{d}},$$

(22) $$\left[{\mathrm{Fe}}^{4+}\right]=\frac{0.9\cdot K_{\mathrm{ox}}T_{s}}{K_{\mathrm{ox}}T_{s}+\sqrt{K_{\mathrm{ox}}T_{s}}+K_{d}},$$

(23) $$\left[O^{2-}\right]=3-\delta -\Delta \delta_{\mathrm{ref}},$$

(24) $$\left[V_{o}\right]=\delta -w-\Delta \delta_{\mathrm{ref}}.$$

The results of the statistical thermodynamic modeling are shown in Figure 6a,b by red lines. Notice that, at high oxygen content in SrFe_0.9_V_0.1_O_3−δ_, the partial molar enthalpy of oxygen corresponds well to the enthalpy of the Fe^3+^ oxidation reaction (Equation (2)). After the breakpoint, ∆*h*_O_ decreases down to the enthalpy of Fe^2+^ oxidation ($\Delta H_{\mathrm{ox}}^{0}$ − 2$\Delta H_{d}^{0}$) in accordance with the reaction $\frac{1}{2}O_{2}+V_{O}+2{\mathrm{Fe}}^{2+}⇄O^{2-}+2{\mathrm{Fe}}^{3+}$. The corresponding levels are shown in Figure 6a by the horizontal dashed lines. This agreement further validates the proposed point defect model.

### 3.7. Nonstoichiometry and Tolerance Factor at Room Temperature

According to TGA results (Figure S2), SrFe_0.9_V_0.1_O_3−δ_ remains oxygen deficient at room temperature when the oxygen content is approximately 2.8. This corresponds to the average oxidation state of iron-site cations of +3.6. As all vanadium in these conditions is pentavalent, the concentrations of Fe^3+^ and Fe^4+^ per formula unit are equal to 0.5 and 0.4, respectively. One may calculate the Goldschmidt tolerance factor [34] as follows:

(25) $$t=\frac{r_{A}+r_{O^{2-}}}{\sqrt{2}(r_{B}+r_{O^{2-}})}$$

where *r*_A_ and *r*_B_ are the average radii of A- and B-cations, respectively, and $r_{O^{2-}}$ is the radius of oxygen anions. The data on ionic radii [35] provide a value of *t* = 1.006, which corresponds to the stability criterion for cubic perovskite structures [34]. Note that even a slightly larger *t* value would lead to structural instability, thus explaining why the solid solution formation limit in an SrFe_1−*x*_V*_x_*O_3−δ_ system corresponds approximately to *x* = 0.1.

### 3.8. Lattice Expansion and Electrical Conductivity

**Figure 7:**
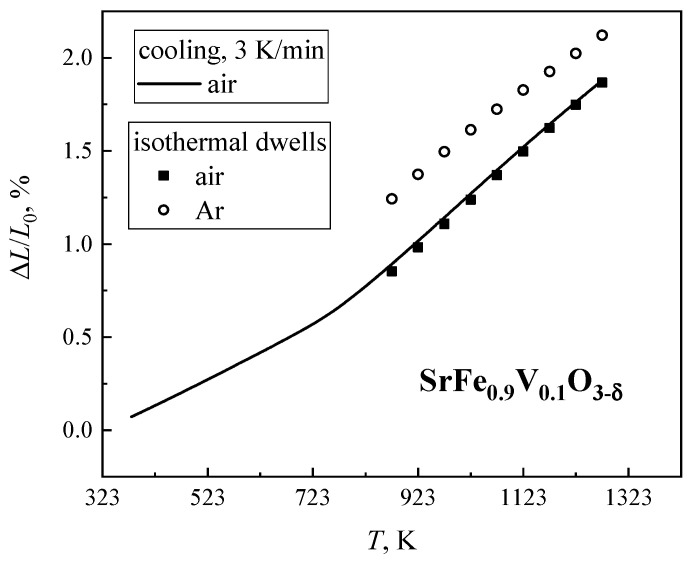
Temperature dependencies of the relative elongations of SrFe_0.9_V_0.1_O_3−δ_ ceramics, collected in the regimes of continuous cooling and isothermal dwells.

**Figure 8:**
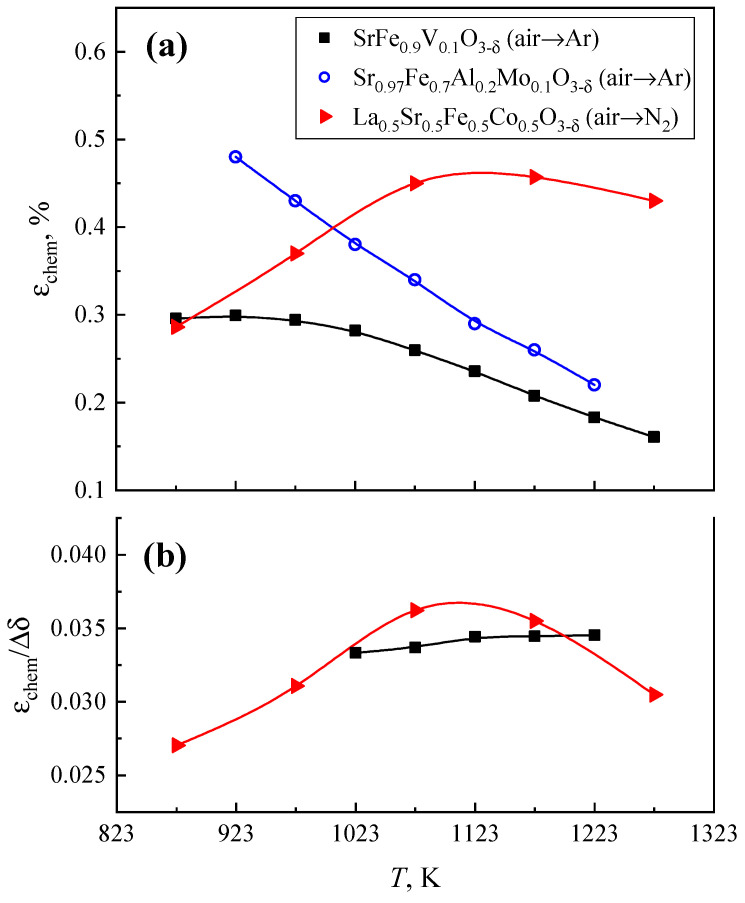
Temperature dependencies of the chemical expansion on isothermal reduction (**a**) and ɛ_chem_/Δδ coefficient (**b**) for SrFe_0.9_V_0.1_O_3−δ_ ceramics. Data on La_0.5_Sr_0.5_Fe_0.5_Co_0.5_O_3−δ_ [36] and Sr_0.97_Fe_0.7_Al_0.2_Mo_0.1_O_3−δ_ [37] are shown for comparison.

The dilatometric curve of SrFe_0.9_V_0.1_O_3−δ_ ceramics, recorded in the dynamic mode in air, exhibits two linear parts with a transition at 780 K when extensive oxygen losses with increasing temperature start (Figure 7). In the low-temperature range, the TEC is equal to (14.2 ± 0.1) × 10^−6^ K^−1^ (Table 2). This value reflects mainly thermal-induced lattice expansion. At higher temperatures, the apparent TEC increases up to (24.8 ± 0.1) × 10^−6^ K^−1^ due to the chemical contribution associated with a decreasing iron oxidation state. Within the limits of experimental uncertainties, the latter value is equal to that obtained using the static regime with isothermal dwells for equilibration at 873–1273 K. During isothermal reduction in flowing argon, an increase in chemical expansion with decreasing temperature is observed (Figure 8a). This behavior well correlates with the oxygen nonstoichiometry variations (Figure 4a). As a consequence, the ɛ_chem_/Δδ coefficient (so-called chemical expansivity) is almost independent of temperature (Figure 8b). Another necessary comment is that the chemical expansion of SrFe_0.9_V_0.1_O_3−δ_ ceramics is moderate with respect to other ferrite-based mixed conductors considered to be promising electrode materials (e.g., [36]). For the sake of comparison, Figure 8a displays analogous data on La_0.5_Sr_0.5_Fe_0.5_Co_0.5_O_3−δ_ [36] and Sr_0.97_Fe_0.7_Al_0.2_Mo_0.1_O_3−δ_ [37] perovskites.

The average TEC of SrFe_0.9_V_0.1_O_3−δ_ ceramics in an argon atmosphere is lower than that in air (Table 2), again in correlation with the *p*(O_2_)-*T*-(3-δ) diagram. Notice that, at elevated temperatures, the average TEC of SrFeO_3−δ_ is as high as (33–34) × 10^−6^ K^−1^ [3,8]. Therefore, doping with 10% vanadium makes it possible to improve thermomechanical properties, a result of a lower oxygen deficiency and lower iron oxidation state in SrFe_0.9_V_0.1_O_3−δ_ compared to SrFeO_3−δ_ (Figure S2). In addition, the TEC of SrFe_0.9_V_0.1_O_3−δ_ is also lower than those for other 10% B-site-doped ferrites, such as SrFe_0.9_Nb_0.1_O_3−δ_ and SrFe_0.9_Ta_0.1_O_3−δ_ (Table 2). At the same time, the expansion of SrFe_0.9_V_0.1_O_3−δ_ in the high temperature range is still larger compared to most SOFC electrolytes based on zirconia, ceria and lanthanum gallate [8]. The chemical contribution to the lattice expansion may have a negative impact on the long-term stability of the electrodes under current and temperature variations, inducing disruptions in the mechanical integrity.

**Figure 9:**
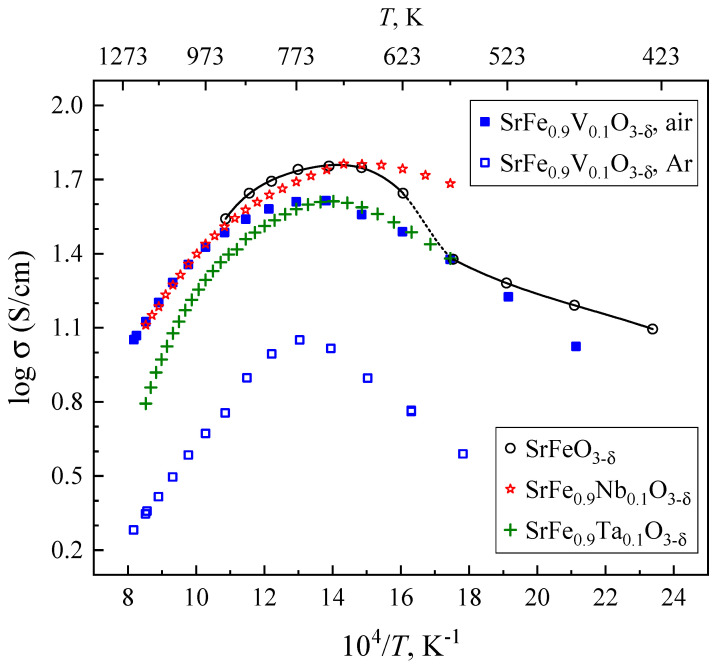
Temperature dependencies of the total electrical conductivity of SrFe_0.9_V_0.1_O_3−δ_ ceramics. Data on SrFeO_3−δ_ [38], SrFe_0.9_Nb_0.1_O_3−δ_ [16] and SrFe_0.9_Ta_0.1_O_3−δ_ [19] in air are shown for the sake of comparison.

The temperature dependencies of total electrical conductivity (Figure 9) also exhibit a behavior typical for perovskite-like ferrites [4,7], where the appearance of maxima at 600–800 K is associated with the progressive release of oxygen from the crystal lattice at high temperatures, as discussed above. The observed decrease in conductivity on heating appears due to the dominant role of *p*-type electronic charge carriers, the concentration of which decreases with increasing oxygen nonstoichiometry. As *p*(O_2_) decreases, the number of oxygen vacancies increases and the concentration of electron holes becomes lower (Figure 4a,b). One should separately mention that the incorporation of vanadium in SrFeO_3−δ_ reduces the concentration of Fe^4+^ and Fe^3+^ states involved in the *p*-type electronic transport (Figure 4b). Moreover, the presence of relatively stable V^5+^ in the iron sublattice leads to a lower concentration of Fe-O-Fe bonds responsible for the electron transfer. Note also that V^5+^ has a smaller ionic radius (0.54 Å) than Fe^3+^ (0.645 Å) [35], which can lead to local lattice distortions. As a result, a moderate decrease in electrical conductivity and an increase in its activation energy at temperatures below 700 K are observed when vanadium is substituted for iron (Figure 9). In the case of SrFe_0.9_V_0.1_O_3−δ_, the apparent activation energy for the conductivity in the low-temperature range is 21 ± 2 kJ/mol in air and 26 ± 1 kJ/mol in an argon atmosphere. At the same time, the conductivity of V-substituted SrFeO_3−δ_ is higher compared to Ta-doped SrFeO_3−δ_ and is nearly equal to that of SrFe_0.9_Nb_0.1_O_3−δ_ at elevated temperatures (Figure 9).

### 3.9. Electrode Behavior

**Figure 10:**
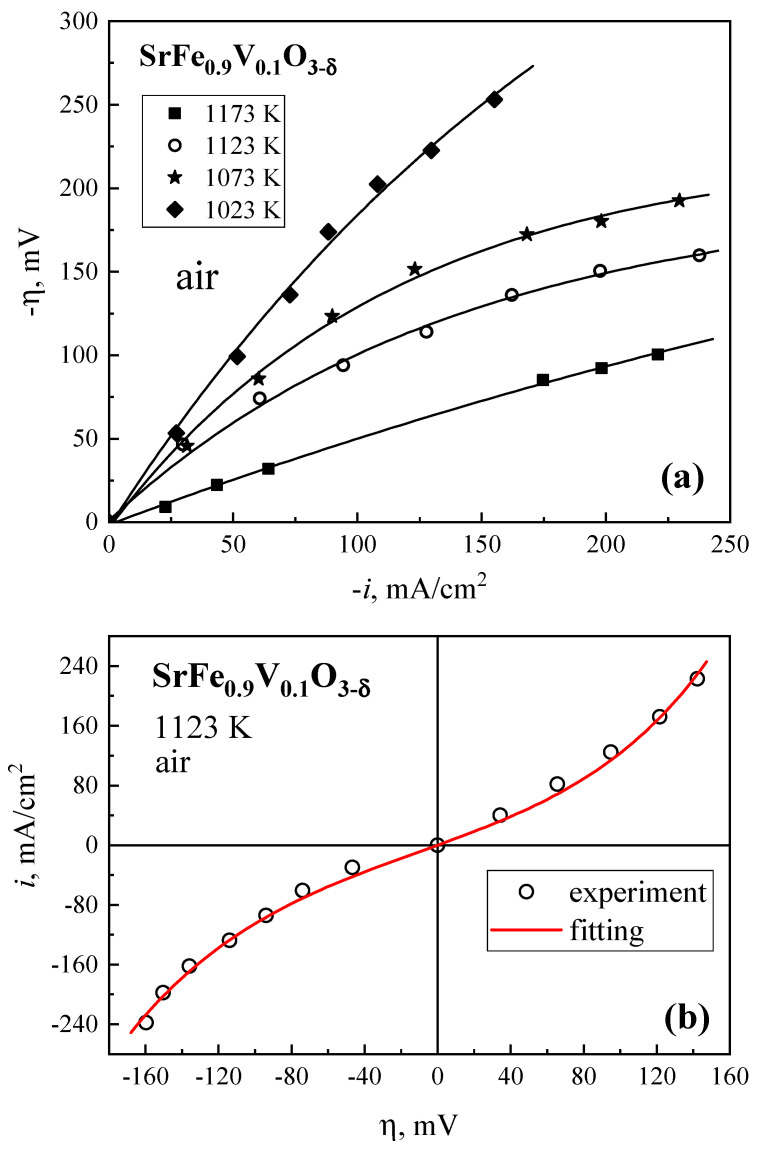
Current density dependencies of the cathodic overpotentials of a porous SrFe_0.9_V_0.1_O_3−δ_ electrode in air at 1023-1173 K (**a**) and of the cathodic and anodic overpotentials at 1123 K (**b**). For (**b**), red solid line shows fitting results.

In order to simulate the operating conditions in SOFC cathodes and SOEC anodes, the electrochemical activity of SrFe_0.9_V_0.1_O_3−δ_ electrodes was tested under current loads in both directions. The polarization curves (Figure 10) exhibit nonlinear behavior; the anodic overpotentials are slightly lower than the absolute values of the cathodic ones. This trend can be explained by increasing oxygen chemical potential at the WE surface under anodic polarization with respect to equilibrium. Such an increase results in a higher concentration of electron holes and electrical conductivity. Cathodic polarization has the opposite effect. These differences mean that the SrFe_0.9_V_0.1_O_3−δ_ electrode demonstrates higher electrochemical activity in electrolysis mode compared to the fuel cell regime. Despite the differences in anodic and cathodic kinetics, the entire polarization curves can be satisfactorily described by the Butler–Volmer equation [39]:

(26) $$i=i_{o}\left[\exp\left(\frac{\alpha nF\eta }{RT}\right)-\exp\left(-\frac{\beta nF\eta }{RT}\right)\right],$$

where *n* is the number of electrons participating in the rate-determining step, α and β are the charge transfer coefficients and *i*_0_ is the exchange current density. One example of the fitting results using this equation is presented in Figure 10b. The calculated *i*_0_ value is 35 ± 5 mA/cm^2^.

**Figure 11:**
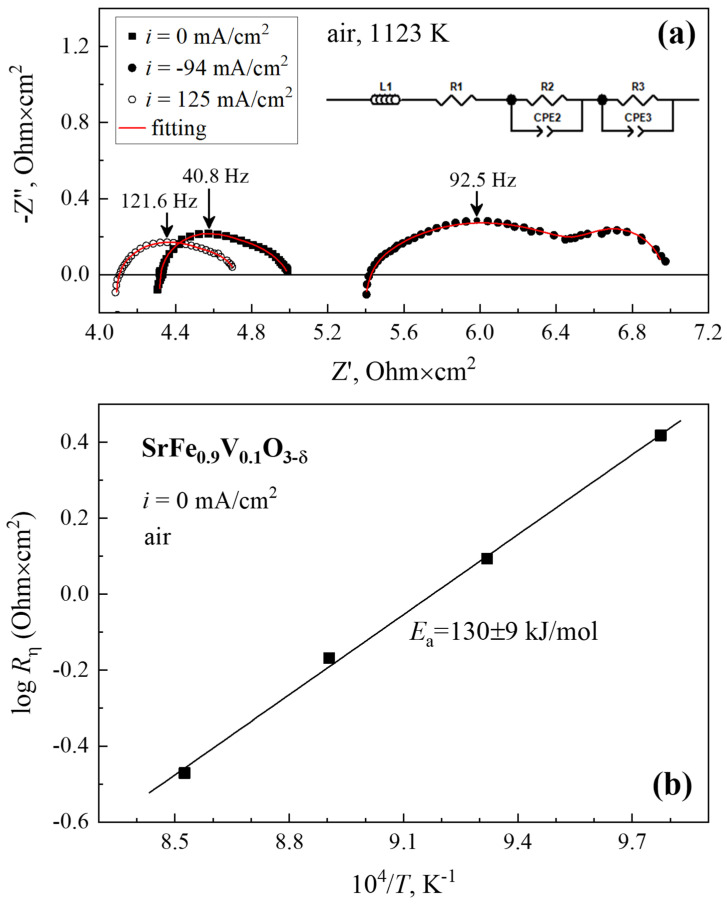
Examples of the impedance spectra for a porous SrFe_0.9_V_0.1_O_3−δ_ electrode (**a**) and temperature dependence of the area-specific polarization resistance under open-circuit conditions (**b**).

The impedance spectra consist of two separable arcs, which were modeled by two sequential processes (Figure 11a). The high-frequency signal was attributed to the charge transfer process, whilst the signal at low frequencies relates to diffusion phenomena. At the cathodic current density of −128 mA/cm^2^, the overpotential is approximately −114 mV at 1123 K. Under the open-circuit conditions, the activation energy for reciprocal electrode polarization resistance is 130 ± 9 kJ/mol (Figure 11b), very close to that obtained for SrFe_0.9_Nb_0.1_O_3−δ_ [16]. This value is typical for the activation energies of oxygen reduction reactions and oxygen diffusion coefficients in La_1−x_Sr_x_Fe_1−y_Co_y_O_3−δ_ electrodes ([40,41] and references therein). The electrochemical activity of the SrFe_0.9_V_0.1_O_3−δ_ electrode is higher compared to a number of its analogues based on perovskite-type ferrite and chromite, in particular La_0.45_Ce_0.05_Sr_0.5_FeO_3−δ_ and (La_0.75_Sr_0.25_)_0.95_Cr_0.5_Mn_0.5_O_3−δ_ studied under similar conditions [25,26]. For further improvement in the electrochemical activity, microstructural optimization and surface modification of the electrodes are necessary in order to increase exchange currents and conductivity.

## 4. Conclusions

XRD analysis of a perovskite-like SrFe_1−*x*_V*_x_*O_3−δ_ system showed that the maximum solid solubility of vanadium at atmospheric oxygen pressure corresponds to approximately 10% iron sites. The Goldschmidt tolerance factor of SrFe_0.9_V_0.1_O_3−δ_, calculated accounting for the oxidation states of the transition metal cations at room temperature in air, is close to unity. Consequently, this phase possesses a cubic perovskite structure (SG *Pm*$\bar{3}$*m*). The *p*(O_2_)-*T*-(3-δ) diagram of SrFe_0.9_V_0.1_O_3−δ_ in the oxygen partial pressure range from 10^−21^ to 0.5 atm at 1023–1223 K can be described by an ideal solution model neglecting any reduction of pentavalent V^5+^ cations within the phase stability limits. This hypothesis was further confirmed by statistical thermodynamic modeling. If compared to undoped SrFeO_3−δ_, the substitution with vanadium leads both to lowering oxygen vacancy concentrations and to decreasing average oxidation states of iron cations. These effects are, in turn, responsible for the observed decrease in *p*-type electronic conductivity, average TECs, and chemical expansion induced by the oxygen nonstoichiometry variations. Porous SrFe_0.9_V_0.1_O_3−δ_ electrodes exhibit relatively good electrochemical activity under oxidizing conditions, which is higher with respect to a number of ferrite- and chromite-based electrode materials. The electrochemical behavior of SrFe_0.9_V_0.1_O_3−δ_ electrodes suggests the possible relevance of electronic conduction as a polarization resistance-affecting factor.

## Figures and Tables

**Table 1:** Thermodynamic parameters (*) of the defect formation reactions in SrFe_0.9_V_0.1_O_3−δ_ and other strontium ferrite-based phases.

Composition	$\Delta H_{ox}^{0}$, kJ·mol^−1^	$\Delta S_{ox}^{0}$, J·mol^−1^·K^−1^	$\Delta H_{d}^{0}$, kJ·mol^−1^	$\Delta S_{d}^{0}$, J·mol^−1^·K^−1^	*w*	Ref.
SrFe_0.9_V_0.1_O_3−δ_	−98.4 ± 0.6	−78.2 ± 0.5	129 ± 1	7.5 ± 0.5	0.203 ± 0.001	this work
SrFe_0.93_Mo_0.07_O_3−δ_	−94 ± 3	−80 ± 2	132 ± 3	7 ± 2	-	[5]
SrFeO_3−δ_	−102 ± 6	−87 ± 5	122.5 ± 0.2	0 **	-	[5]

* The temperature ranges used for the calculations were 1023–1223 K for SrFe_0.9_V_0.1_O_3−δ_, 1073–1223 K for SrFe_0.93_Mo_0.07_O_3−δ_ and 1148–1223 K for SrFeO_3−δ_. ** Statistically insignificant.

**Table 2:** Apparent TECs of SrFeO_3−δ_-based materials.

Composition	Atmosphere	Conditions	*T*, K	TEC × 10^6^, K^−1^	Ref.
SrFe_0.9_V_0.1_O_3−δ_	air	isothermal dwells	873–1273	25.0 ± 0.3	This work
		cooling, 3 K/min	873–1273	24.8 ± 0.1	
			373–673	14.2 ± 0.1	
	Ar *	isothermal dwells	873–1273	22 ± 1	
SrFe_0.9_Nb_0.1_O_3−δ_	air	heating, 5 K/min	683–1273	27.5	[16]
			300–683	15.6	
SrFe_0.9_Ta_0.1_O_3−δ_	air	heating	823–1173	37.0	[18]
			300–823	14.8	
SrFeO_3−δ_	air	heating, 2 K/min	623–1373	34.1	[3]
			323–523	15.6	

* *p*(O_2_) = 7.6 × 10^−5^ atm.

## Data Availability

The original contributions presented in the study are included in the article, further inquiries can be directed to the corresponding authors.

## Supplementary Materials

The following supporting information can be downloaded at https://www.mdpi.com/article/10.3390/ma18030493/s1, Figure S1: Illustration of the static regime of the dilatometric measurements in flowing air and Ar on temperature cycling at 873–1273 K with equilibration at each temperature; Figure S2: Oxygen content in SrFe_0_._9_V_0_._1_O_3−δ_ as a function of temperature in air. Data on SrFeO_3−δ_ [5] and SrFe_0_._9_Nb_0_._1_O_3−δ_ [42] are shown for the sake of comparison.

## Nomenclature

List of abbreviations	
10Sc1YSZ	10 mol.% scandia- and 1 mol.% yttria-co-stabilized zirconia
CE	counter electrode
DC	direct current
EDX	energy-dispersive X-ray spectroscopy
GDC10	Ce_0.9_Gd_0.1_O_2−δ_
GOF	goodness of fit
RE	reference electrode
SEM	scanning electron microscopy
SG	space group
SOFC	solid oxide fuel cell
SOEC	solid oxide electrolysis cell
TEC	average linear thermal expansion coefficient
TGA	thermogravimetric analysis
WE	working electrode
XRD	X-ray diffraction
List of symbols	
*a*	unit cell parameter
*F*	Faraday constant
G	total Gibbs energy
$G^{0}$	standard Gibbs energy
$\Delta G_{i}^{0}$	standard free Gibbs energy of defect formation reaction
$\Delta H_{i}^{0}$	enthalpy of defect formation reaction
∆*h*_O_	enthalpy of oxygen incorporation in the lattice
*i*	current density
*i*_0_	exchange current density
*K*_d_	equilibrium constant of charge disproportionation reaction
*K*_e_	equilibrium constant of electron exchange reaction
*K*_ox_	equilibrium constant of iron oxidation reaction
*n*	number of electrons participating in the rate-determining step
*p*(O_2_)	oxygen partial pressure
*R*	molar gas constant
*r*_A_	average radius of A-cation
*r*_B_	average radius of B-cations
*R*_η_	polarization resistance
$r_{O^{2-}}$	radius of oxygen anion
$S_{\mathrm{conf}}$	total configurational entropy
$\Delta S_{i}^{0}$	entropy of defect formation reaction
∆*s*_O_	entropy of oxygen incorporation in the lattice
*T*	absolute temperature
*t*	Goldschmidt tolerance factor
*w*	number of anion sites unavailable for oxygen ions
wR	weighted profile R-factor
wR_min_	expected R-factor
α, β	charge transfer coefficients
δ	oxygen nonstoichiometry
Δδ_ref_	parameter for correction of experimental data on the oxygen content
ε_chem_	isothermal chemical expansion
η	overpotential
θ	diffraction angle
$\mu_{i}^{0}$	standard chemical potential
∆μ_O_	oxygen chemical potential
σ	total electrical conductivity
