# Supplementary material for: Oxygen Nonstoichiometry, Electrical Conductivity, Chemical Expansion and Electrode Properties of Perovskite-Type SrFe0.9V0.1O3−δ
Source: Materials (Basel). 2025 Jan 22;18(3):493. doi: 10.3390/ma18030493 (PMC11818172; doi:10.3390/ma18030493)
Supplement: Supplementary file 1 [file materials-18-00493-s001.zip › materials-3242910-supplementary.pdf]

Supplementary materials

Oxygen Nonstoichiometry, Electrical Conductivity, Chemical Expansion and Electrode Properties of Perovskite-Type $SrFe_{0.9}V_{0.1}O_{3-\delta}$

Aleksei I. Ivanov [1,*], Sergey S. Nikitin [1], Mariya S. Dyakina [1], Ekaterina V. Tsipis [1,*], Mikhail V. Patrakeev [1], Dmitrii A. Agarkov [1,2], Irina I. Zverkova [1], Andrey O. Zhigachev [1], Victor V. Kedrov [1] and Vladislav V. Kharton [1]

[1] Osipyan Institute of Solid State Physics RAS, Chernogolovka 142432, Russia

[2] Moscow Institute of Physics and Technology, Dolgoprudny 141701, Russia

*Correspondence: aliv@issp.ac.ru (A.I.I.); tsipis@issp.ac.ru (E.V.T.)

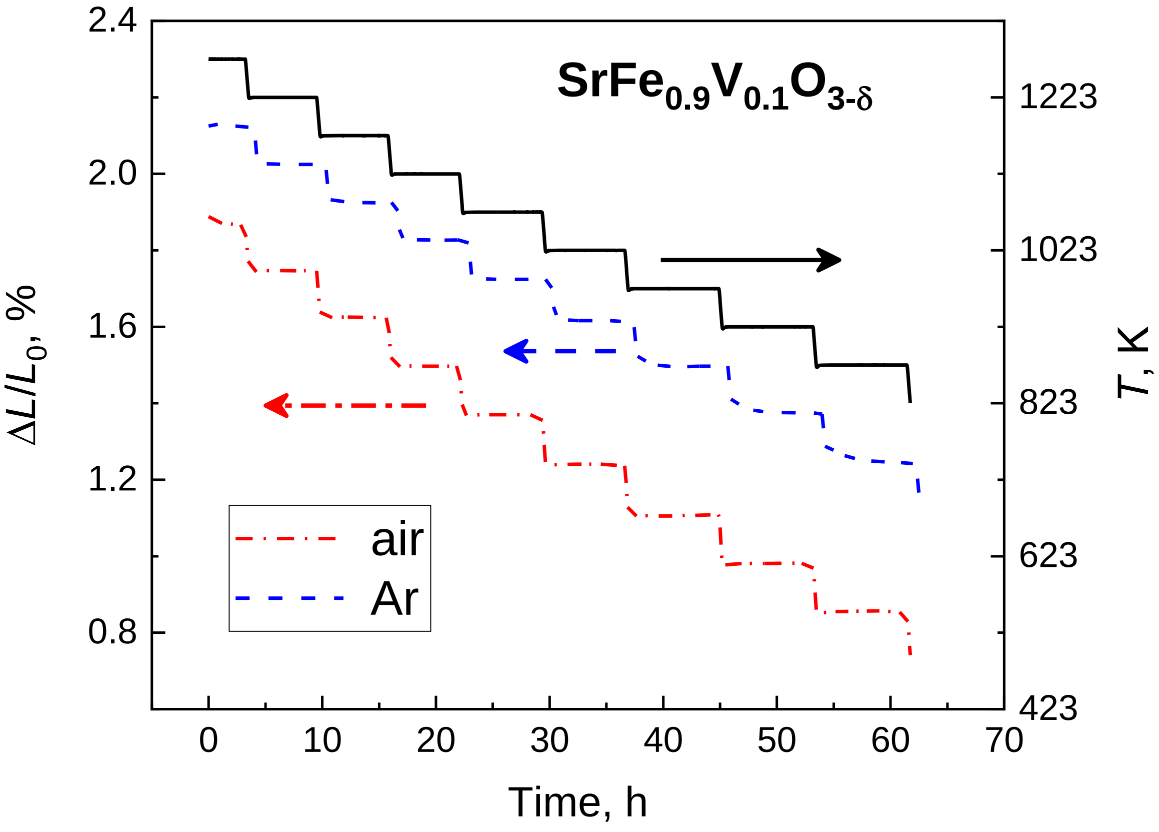

Fig. S1. Illustration of the static regime of the dilatometric measurements in flowing air and Ar on temperature cycling at 873-1273 K with equilibration at each temperature.

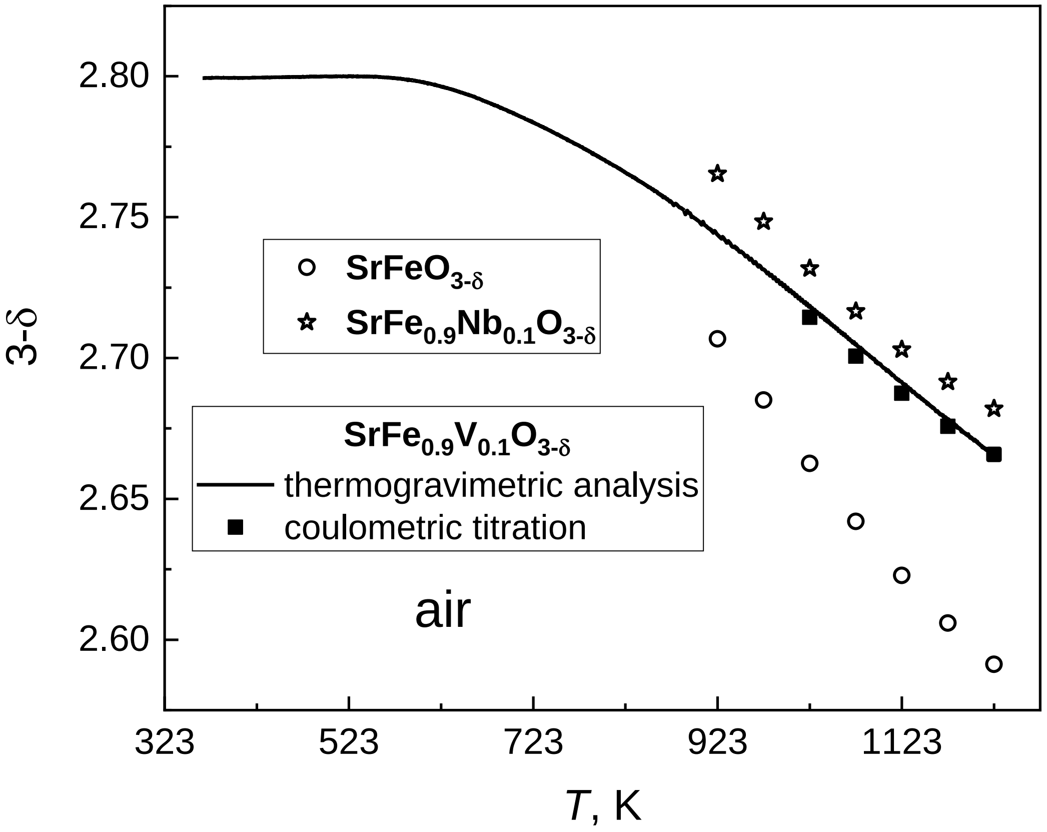

Fig. S2. Oxygen content in $SrFe_{0.9}V_{0.1}O_{3-\delta}$ as a function of temperature in air. Data on $SrFeO_{3-\delta}$ [5] and $SrFe_{0.9}Nb_{0.1}O_{3-\delta}$ [42] are shown for the sake of comparison.
